# A Remote Digital Monitoring Platform to Assess Cognitive and Motor Symptoms in Huntington Disease: Cross-sectional Validation Study

**DOI:** 10.2196/32997

**Published:** 2022-06-28

**Authors:** Florian Lipsmeier, Cedric Simillion, Atieh Bamdadian, Rosanna Tortelli, Lauren M Byrne, Yan-Ping Zhang, Detlef Wolf, Anne V Smith, Christian Czech, Christian Gossens, Patrick Weydt, Scott A Schobel, Filipe B Rodrigues, Edward J Wild, Michael Lindemann

**Affiliations:** 1 Roche Pharma Research and Early Development, pRED Informatics, Pharmaceutical Sciences, Clinical Pharmacology, and Neuroscience, Ophthalmology, and Rare Diseases Discovery and Translational Area Roche Innovation Center Basel F. Hoffmann-La Roche Ltd Basel Switzerland; 2 Huntington’s Disease Centre UCL Queen Square Institute of Neurology University College London London United Kingdom; 3 Ionis Pharmaceuticals Inc Carlsbad, CA United States; 4 Rare Disease Research Unit Pfizer Nice France; 5 Department of Neurology University of Ulm Medical Center Ulm Germany; 6 Department of Neurodegenerative Disease and Gerontopsychiatry/Neurology University of Bonn Medical Center Bonn Germany; 7 F. Hoffmann-La Roche Ltd Basel Switzerland

**Keywords:** Huntington disease, digital monitoring, digital biomarkers, remote monitoring, smartphone, smartwatch, cognition, motor, clinical trials, mobile phone

## Abstract

**Background:**

Remote monitoring of Huntington disease (HD) signs and symptoms using digital technologies may enhance early clinical diagnosis and tracking of disease progression, guide treatment decisions, and monitor response to disease-modifying agents. Several recent studies in neurodegenerative diseases have demonstrated the feasibility of digital symptom monitoring.

**Objective:**

The aim of this study was to evaluate a novel smartwatch- and smartphone-based digital monitoring platform to remotely monitor signs and symptoms of HD.

**Methods:**

This analysis aimed to determine the feasibility and reliability of the Roche HD Digital Monitoring Platform over a 4-week period and cross-sectional validity over a 2-week interval. Key criteria assessed were feasibility, evaluated by adherence and quality control failure rates; test-retest reliability; known-groups validity; and convergent validity of sensor-based measures with existing clinical measures. Data from 3 studies were used: the predrug screening phase of an open-label extension study evaluating tominersen (NCT03342053) and 2 untreated cohorts—the HD Natural History Study (NCT03664804) and the Digital-HD study. Across these studies, controls (n=20) and individuals with premanifest (n=20) or manifest (n=179) HD completed 6 motor and 2 cognitive tests at home and in the clinic.

**Results:**

Participants in the open-label extension study, the HD Natural History Study, and the Digital-HD study completed 89.95% (1164/1294), 72.01% (2025/2812), and 68.98% (1454/2108) of the active tests, respectively. All sensor-based features showed good to excellent test-retest reliability (intraclass correlation coefficient 0.89-0.98) and generally low quality control failure rates. Good overall convergent validity of sensor-derived features to Unified HD Rating Scale outcomes and good overall known-groups validity among controls, premanifest, and manifest participants were observed. Among participants with manifest HD, the digital cognitive tests demonstrated the strongest correlations with analogous in-clinic tests (Pearson correlation coefficient 0.79-0.90).

**Conclusions:**

These results show the potential of the HD Digital Monitoring Platform to provide reliable, valid, continuous remote monitoring of HD symptoms, facilitating the evaluation of novel treatments and enhanced clinical monitoring and care for individuals with HD.

## Introduction

### Background

Huntington disease (HD) is a genetic, neurodegenerative, and ultimately fatal disease characterized by a triad of cognitive, behavioral, and motor symptoms leading to functional decline and progressive loss of independence [1,2]. The clinical assessment of HD primarily relies on periodic in-person clinical assessments and may include administration of clinician-rated outcomes (which are dependent on rater experience and expertise) or patient-reported outcomes [3,4]. The infrequency of these assessments can result in subtle changes in cognition, behavior or motor abilities being unnoticed, and fluctuations in signs and symptoms being undetected [3,5]. Moreover, in-clinic assessments of disease symptoms that affect patients’ daily experiences are removed from the daily context in which patients experience these symptoms [3]. Taken together, there is a need for improvement in the monitoring of HD signs, symptoms, and functional impacts to enhance accurate characterization of the clinical course and detection of treatment effects.

Recent studies have demonstrated the feasibility and initial validation of wearable sensors as objective measures of HD motor symptoms in the home setting [6-10]. Acquired sensor data on motor function differentiated individuals with HD from control participants, as well as individuals with HD grouped by motor impairment as measured by the Unified HD Rating Scale-Total Motor Score (UHDRS-TMS) [6]. Additionally, sensor data revealed disease features not observed during in-clinic assessments, such as an increased proportion of time spent lying down among participants with HD who were ambulatory compared with control participants [7,9]. A pilot study of a smartphone app in 23 participants showed a significant difference in chorea score and tap rate between individuals with and without manifest HD [11]. Furthermore, the digital measure of tap rate strongly correlated with the UHDRS finger tapping score [11]. Finally, the feasibility and validity of a smartphone app for remote assessment of HD cognitive measures were evaluated in a study of 42 participants. The study found that the digital cognitive tests had robust test-retest reliability for participants with manifest HD (intraclass correlation coefficient [ICC] 0.71-0.96) [12]. Correlations between the digital cognitive tasks and selected Enroll-HD cognitive tasks varied in strength (*r*=0.36-0.68) [12]. Despite these promising exploratory findings in HD and favorable results from digital platforms for Parkinson disease [13-21], to date, there have been no formal validation efforts of an at-home digital-based monitoring system that includes assessments for both motor and nonmotor symptoms of HD.

Although most studies on digital measures of neurodegenerative diseases have focused on motor symptom assessments, a more comprehensive assessment of function that includes both motor and nonmotor outcomes is needed to provide a more holistic disease characterization. Furthermore, digital platforms that include a combination of motor and nonmotor active tests, passive monitoring of daily activities, and patient-reported outcomes can generate data that can be interpreted as being meaningful to patients. Indeed, previous studies have demonstrated that gait and balance impairments increase fall risk and greatly influence the quality of life of people with HD [22]. Furthermore, the cognitive and neuropsychiatric characteristics of HD contribute greatly to the loss of functional independence and quality of life, and hence require evaluation [23-26].

### This Study

In this study, a smartwatch- and smartphone-based remote digital monitoring platform was developed to assess motor, cognitive, behavioral, and functional domains in HD using frequent active and continuous passive monitoring [27,28]. This platform was applied to individuals with premanifest HD (individuals genetically confirmed to have HD but not having diagnostic motor symptoms of HD), manifest HD (individuals with diagnostic motor symptoms of HD), and control participants to determine its feasibility, reliability, and cross-sectional validity for monitoring motor and cognitive features, which are key domains that change with clinical progression across the continuum of adult HD [1]. Digital-based outcomes were compared at baseline with analogous in-clinic tests during the screening period from 3 independent studies (a recently completed open-label extension [OLE; NCT03342053] of a tominersen phase I/IIa study and 2 untreated natural history cohorts: the HD Natural History Study [NHS; NCT03664804] and the University College London Digital-HD study) to cross-sectionally validate the Roche HD Digital Monitoring Platform, with results reported herein.

## Methods

### Study Design and Setting

The analysis sample included pretreatment data from the OLE of a tominersen phase I/IIa study and 2 untreated natural history cohorts: HD NHS and the University College London Digital-HD study. The OLE study was designed to assess the safety, tolerability, pharmacokinetics, and pharmacodynamics of tominersen in patients with manifest HD as well as its effects on digital and standard clinical measures. The OLE study included a 4-week screening period before the start of the treatment period. The HD NHS was designed to evaluate the relationship between changes in cerebrospinal fluid mutant huntingtin protein levels and clinical outcomes in untreated patients with manifest HD. A 4-week screening period was included in the HD NHS. Digital-HD was an observational study that evaluated the tolerability and feasibility of conducting smartphone- and smartwatch-based remote patient monitoring in HD over 18 months.

In this study, we report an analysis of sensor-based outcomes from the 3 studies to assess the cognitive and motor domains. Data from the first 4 weeks after issuing the remote monitoring devices to the participants were considered for the analysis; for the OLE study and the HD NHS, this included only data collected during the 4-week screening periods, up to the baseline assessment. Adherence metrics were collected upon deployment of the digital devices to participants. As part of the study setup for the digital monitoring platform solution, adherence monitoring and processes for follow-up were implemented in case of drops in participant adherence. However, there were no incentives, financial or otherwise, for high adherence, nor did poor adherence lead to exclusion of the participant from the study. The longitudinal effects of tominersen on digital outcomes acquired from the OLE study are not the focus of this study and hence not reported here.

### Participants

All participants enrolled in each respective study were eligible for this analysis. Written, informed consent was obtained from all participants. To be eligible for the OLE study, patients must have completed the treatment period of the phase I/IIa study. Patients in the phase I/IIa study had early manifest HD, Shoulson-Fahn stage I disease (UHDRS-Total Functional Capacity [TFC] score 11-13). Participants in the HD NHS had early manifest HD, Shoulson-Fahn stage I/II disease (UHDRS-TFC score 7-13). Participants from the phase I/IIa study (N=46) and the HD NHS (N=94) were aged 25 to 65 years. The Digital-HD study (N=79) enrolled adults (aged 18 to 75 years) with manifest HD (diagnostic confidence level=4, stage I-III, UHDRS-TFC 4-13, cytosine adenine guanine [CAG] expansion≥36), premanifest HD (diagnostic confidence level<4, CAG expansion≥40), and healthy control participants (no known family history or CAG expansion<36).

### In-Clinic Assessments

All in-clinic assessments were performed at the screening visit. Clinical signs and symptoms were assessed by trained raters using the UHDRS [29]. The scale assesses 4 domains associated with HD: motor function, cognitive function, behavioral abnormalities, and functional capacity. To assess motor performance, the UHDRS Maximal Chorea item, UHDRS Finger Taps item, and UHDRS-TMS were used. In addition, an in-clinic balance score was generated by summing the UHDRS-TMS Retropulsion Pull test item and UHDRS-TMS Tandem Walking test item scores.

To assess cognitive performance, the Stroop Word Reading (SWR) [30] and the Symbol Digit Modalities Test (SDMT) [31] were used. The SWR is a measure of attention and psychomotor speed and relies on verbal motor output and ability to articulate words. The SDMT was used to assess attention, visuoperceptual processing, working memory, and psychomotor speed. The Speeded Tapping test [32] was applied to measure bradykinesia and motor timing. In this computerized test, participants were instructed to tap on the mouse key as fast as possible for 30 seconds using their index finger. The mouse was fixed on the mouse platform and placed on the table.

### Digital Monitoring Hardware

**Figure 1:**
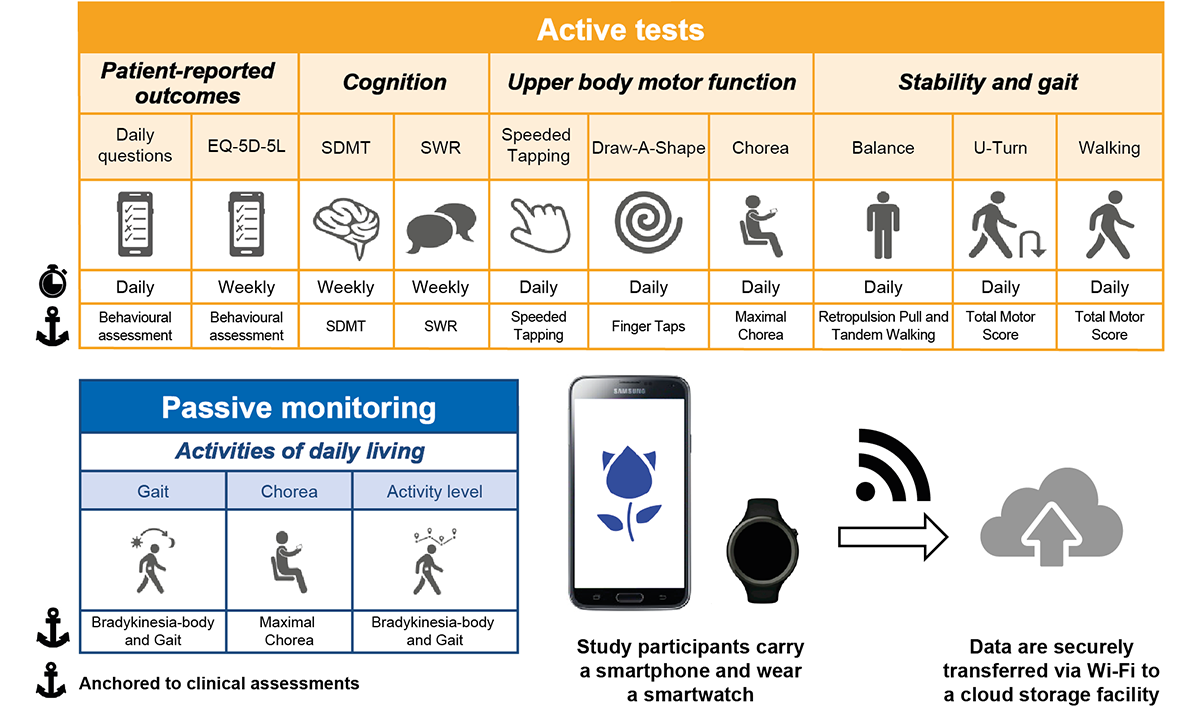
Overview of the Roche HD monitoring app and workflow for the daily assessments. The smartphone (Galaxy J7; Samsung) and smartwatch (Moto G 360 2nd Gen Sport; Motorola) were provided with a preinstalled custom app (Roche HD monitoring app version 1; Roche). Participants were instructed to carry the smartphone in their trouser pocket, or a belt containing a pouch around the waist and wear the smartwatch. The app requested the completion of active tests daily and subsequently recorded sensor data during daily living (passive monitoring). EQ-5D-5L: EuroQol 5-dimension 5-level; SDMT: Symbol Digit Modalities Test; SWR: Stroop Word Reading.

Participants were provided with a wrist-worn smartwatch (Moto G 360 2nd Gen Sport; Motorola), a smartphone (Galaxy J7; Samsung), and a belt containing a pouch to carry the smartphone. Participants received training on their use at the screening visit, at which time the devices were deployed for remote continuous monitoring (Figure 1). The devices were locked and configured to only collect assessments in this study and contained no additional functionality.

### Active Digital Tests

A novel Android app (Roche HD monitoring app version 1) was designed and installed on both the smartphone and smartwatch to measure HD clinical features. Participants were asked to complete specific tests using the devices (*active tests*) and then to carry the devices with them as they conducted their daily routine, during which sensor data were recorded continuously for *passive monitoring* of gait, chorea, and activity level. Results from patient-reported outcomes and passive monitoring are not reported here. Table 1 describes the active tests included in the app and a video depicting the tests can be found in Multimedia Appendix 1. Participants were prompted by the smartphone to complete active tests daily except SWR, SDMT, and EuroQol 5-dimension 5-level, which were completed weekly. Each test was preceded by an instruction screen that named and explained the task (Multimedia Appendix 2).

The digital SDMT assessed the participant’s attention, visuoperceptual processing, working memory, and psychomotor speed by recording the participant’s performance in tapping a number corresponding to a symbol shown on the smartphone screen. A total of 3 sets of symbol-digital mappings were used, and the symbol sequence was fixed for all tests. For a given symbol, participants were required to match it with a 1- to 9-digit using the keypad. The number of correct answers was defined as the number of matching events.

The digital SWR test assessed the participant’s attention, psychomotor speed, and ability to articulate words by recording the participant’s performance in reading color names out loud, row by row, as fast as possible. Names of colors were displayed in black on the screen in a randomly generated sequence (4 words per row and a total of 60 words). Spoken words were automatically recognized by a custom-written word recognizer. The custom-written word recognizer was validated on >30 annotated Stroop tests per language spanning the whole range of severity as defined by the in-clinic SWR test. The number of correctly read words was defined as the number of matching words.

The Speeded Tapping test assessed fine motor impairment by recording the participant’s performance in tapping one button on the screen as fast and as regularly as possible.

The Draw-A-Shape test assessed visuomotor coordination and fine motor impairment by recording the participant’s performance in tracing a series of increasingly complex shapes on the smartphone screen.

The Chorea test captured the degree of chorea by recording upper body physical movements as the seated participant held the smartphone as still as possible in their outstretched arm and hand while wearing the smartwatch on the preferred wrist. As a dual task, the participant counted backward aloud during the test; these data are not included in these analyses as the methodologies needed to analyze these data are still under development.

The Balance test assessed the participant’s static balance function by recording movements as the participant stood as still as possible while wearing the smartphone and smartwatch.

The U-Turn test was designed to assess gait and lower body bradykinesia. The participant walked and turned at least five times between 2 points that were at least four steps apart while wearing the smartphone and smartwatch.

The Walk test captured elements of gait, body bradykinesia, and tandem walking abnormalities. The participant walked as fast as was safely possible for 200 meters or 2 minutes.

The inertial measurement unit (accelerometer, gyroscope, or magnetometer) captured continuous measurements from the smartphone and smartwatch during active tests. The digital SWR and Chorea tests were captured using the microphone in addition to inertial measurement unit recordings. For the digital SDMT, Speeded Tapping, and Draw-A-Shape tests, touchscreen events were recorded. For the digital SDMT, actual answers with timestamps were recorded. Participants were instructed to carry the phone in the provided pouch for the Balance, U-Turn, and Walking tests and in the provided pouch or their trouser pocket for passive monitoring.

**Table 1:** Descriptions of the active tests included in the Roche HD monitoring app version 1.

Domain and test		Description
**Cognition**		
	SDMT^a^	Digital version of the pen-and-paper SDMT; tap the number corresponding to a symbol shown on the screen as fast as possible for 90 seconds
	SWR^b^	Modified digital version of the pen-and-paper SWR; read the color names out loud, row by row, as fast as possible for 45 seconds
**Upper body motor function**		
	Speeded Tapping^c^	Repeatedly tap a virtual button as fast as possible for 30 seconds with the phone flat on a surface
	Draw-A-Shape^c^	Trace a series of reference shapes (diagonal lines, square, circle, figure of eight, or spiral) on the screen with the index finger as quickly and accurately as possible with the phone flat on a surface
	Chorea^c^	Hold phone in the palm of the hand and keep arm outstretched for 30 seconds while keeping eyes closed and counting backward aloud in sevens from a random number shown on the smartphone screen
**Stability and gait**		
	Balance	Stand upright as still as possible for 30 seconds with arms hanging loosely by the sides and phone in waist pouch
	U-Turn	Walk between two points, at least four steps apart, and turn 180 degrees at least five times with the phone in waist pouch during a 60-second period
	Walking	Walk as fast as safely possible for 200 meters or 2 minutes, with phone in waist pouch. Ideally, the test was performed in a straight path with no obstacles

^a^SDMT: Symbol Digit Modalities Test.

^b^SWR: Stroop Word Reading.

^c^Tests are repeated for each hand.

### Data Transfer and Processing

Participants received instructions on how to connect the smartphone to the internet at home. For participants with no wireless internet connection (Wi-Fi) at home, data were uploaded during clinic visits. All data were encrypted and uploaded to secure servers each time the smartphone was connected to Wi-Fi.

### Digital Test Outcomes

The raw data for each test were converted into a single predefined readout, hereafter referred to as a feature. The values reported here are the medians for each feature over 2-week intervals. If, for a participant, less than n observations that passed the quality criteria (*Statistical Analysis*) for a given test in an interval were available, the data for that participant and interval were considered as missing. The value of n was 1 for the SDMT and SWR test and 3 for all other tests. The interval length of 2 weeks was found to be the optimal trade-off between period length and robustness against missing values. The following active test features were prospectively selected based on their face validity as tests of relevant cognitive and motor function in HD and digital surrogates of existing in-clinic tests: number of correct answers (SDMT), number of correctly read words (SWR), and mean intertap interval (Speeded Tapping). The rationale behind the intertap interval is to assess the time that the finger is in the air (not on the glass), as we hypothesized that uncontrolled movements would influence this time span. For all other tests, features were prospectively chosen based on previous literature and their relevance to HD: sway path (Chorea and Balance) [33,34], spiral drawing speed variability (Draw-A-Shape), median turn speed (U-Turn) and step frequency variance (Walking) [35]. The sway path feature offers a straightforward way to measure the amount of movement occurring when a study participant is trying to hold the body or hand as still as possible; this feature has been successfully used in other disease areas in the same context [36]. The median turn speed feature has shown good performance in Parkinson disease and multiple sclerosis [14,37]. This feature is influenced by gait and postural instability problems, which are both prevalent symptoms in HD. Therefore, it was hypothesized that turn speed would measure relevant HD signals. A meta-analysis showed that variability in gait parameters was increased among patients with HD compared with healthy controls, even in comparison with patients with other neurological disorders [38]. These results and expert input led to the decision to select step frequency variance as a feature to measure gait variability while being algorithmically as robust as possible. Variability measures, in general, seem to be sensitive in detecting disease-relevant signals in the upper limb domain also, as has been shown for a tapping test [39]. Although the Draw-A-Shape test offers a plethora of different features to select from, a feature that measures variability, drawing speed variability (as measured by the coefficient of variation of drawing speed, for the shape where we expected to see the biggest challenges in maintaining drawing speed, ie, the spiral) was chosen.

### Statistical Analysis

As a quality control (QC) measure, active tests were excluded via quality criteria assessing the correct test execution (Multimedia Appendix 3). Overall adherence is reported as the proportion of tests completed during the 4-week study period.

Test-retest reliability of active test feature data from participants was calculated using the ICC [40] between the median values of the first 2 weeks and those of the second 2 weeks, and occurred predrug exposure in the OLE study.

To investigate convergent validity (ie, the degree to which 2 measures of the same construct are related) of sensor-derived features, Spearman correlation was calculated between clinical scores acquired at baseline visit and sensor-derived features that were median-aggregated over the first 2 weeks of data collection. Pearson correlation was used when both variables were normally distributed and a linear relationship between them was expected to exist. All analyses were conducted with Python (version 3.6; Python Software Foundation) scripts using the pandas and SciPy libraries.

Known-groups validity was assessed by comparing the feature values between the 3 cohorts from the Digital-HD study (premanifest HD, manifest HD, and controls) and the 2 manifest HD cohorts from the OLE study and HD NHS. Comparison was done by first fitting for each value a mixed linear effect model with a fixed effect for age and a random intercept for study. The residuals of this model were then compared using Kruskal-Wallis test and pairwise Mann-Whitney *U* tests between these 5 groups.

### Ethics Approval

All studies were approved by the respective local ethics committees and conducted in accordance with the Declaration of Helsinki and the International Conference on Harmonisation Guidelines for Good Clinical Practice.

The OLE study protocol was approved by the following ethics committees: National Research Ethics Service Committee London-West London and GTAC, London, United Kingdom; Ethik-Kommission der Medizinischen Fakultät der Universität Ulm, Germany; and University of British Columbia Clinical Ethics Review Board, Canada. The HD NHS protocol was approved by the following ethics committees: The University of British Columbia Clinical Research Ethics Office, Vancouver, British Columbia, Canada; Advarra, Aurora, Ontario, Canada; Universität Ulm – Ethik - Kommission, Germany; Ethik - Kommission der Med. Fakultät der Ruhr Universität Bochum, Germany; London - Camden & Kings Cross Research Ethics Committee, London, United Kingdom; Advarra, Columbia, Maryland, United States; University of California Davis Institutional Review Board (IRB) Administration, Sacramento, California, United States; Institutional Review Board, Columbia University Medical Center, New York, New York, United States; Johns Hopkins Medicine Office of Human Subjects Research-IRB East Baltimore Campus, Baltimore, Maryland, United States; HealthOne IRB, Denver, Colorado, United States; Georgetown University Institutional Review Board, Washington DC, United States; and NHS Health Research Authority/Research Ethics Service, London, United Kingdom. The Digital-HD study protocol was approved by London-Central Research Ethics Committee, London, United Kingdom.

### Code Availability

To collect at-home and in-clinic digital data (active tests of cognitive and motor performance, passive monitoring of daily life, and electronic patient-reported outcomes), we relied on custom Android apps built specifically for these studies deployed on smartphones and smartwatches. All other data for the studies (including demographics and clinical scores) were collected manually without the use of software code.

Raw data signal processing (feature extraction) and data analysis (to compute descriptive statistics for demographic variables, implement test-retest reliability, and for group comparisons and correlations) were carried out with Python. The code used to complete the analysis can be made available upon request.

## Results

### Participants

In the OLE study and HD NHS, no participants were lost to follow-up or withdrew during the 4-week period after receiving the digital monitoring equipment, allowing all to be included in the planned test-retest evaluation. In the Digital-HD study, one participant withdrew from the study. Participants’ key baseline demographic and clinical characteristics are provided in Table 2.

**Table 2:** Baseline characteristics of participants in the open-label extension (OLE) study, the Huntington disease Natural History Study (HD NHS), and the Digital-HD study.

Characteristics		OLE study (N=46)	HD NHS (N=94)	Digital-HD study		
				Healthy control (N=20)	Premanifest HD (N=20)	Manifest HD (N=39)
Age (years), mean (SD)		48.6 (10.2)	48.2 (9.9)	48.0 (13.8)	44.9 (10.0)	56.3 (11.0)
**Gender, n (%)**						
	Male	28 (61)	58 (62)	13 (65)	10 (50)	21 (54)
	Female	18 (39)	36 (38)	7 (35)	10 (50)	18 (46)
Number of CAG^a^ repeats, mean (SD)		44.3 (3.0)	44.2 (3.1)	N/A^b^	41.6 (2.0)	42.7 (3.3)^c^
Right hand dominance (laterality), n (%)		43 (93)	81 (86)	17 (85)	20 (100)	29 (74)
TMS^d^, mean (SD)		23.6 (12.5)	22.1 (10.9)	1.4 (2.4)	4.9 (3.9)	32.9 (16.6)
TFC^e^, mean (SD)		11.2 (1.6)	11.0 (1.5)	13.0 (0.0)	12.9 (0.3)	10.6 (2.2)
SWR^f^, mean (SD)		74.0 (21.9)	72.2 (20.0)	100.1 (19.5)	102.3 (19.0)	67.5 (19.4)
SDMT^g^, mean (SD)		33.7 (12.1)	32.3 (11.6)	62.1 (8.4)^h^	56.4 (12.2)^h^	29.0 (12.2)^h^

^a^CAG: cytosine adenine guanine.

^b^N/A: not applicable.

^c^Number of CAG repeats for 3 participants with manifest HD in Digital-HD study not available.

^d^TMS: Total Motor Score.

^e^TFC: Total Functional Capacity.

^f^SWR: Stroop Word Reading.

^g^SDMT: Symbol Digit Modalities Test.

^h^Reported data are an average of 68 participants, as data from 11 participants were discarded owing to tests conducted in 45 seconds rather than 90 seconds.

### QC of Digital Active Test Execution

For the SDMT, Chorea, Speeded Tapping, and SWR tests, a low proportion (ranging between 0% and 6.7% [Digital-HD, healthy controls: 16/238 (6.7%) Chorea—nondominant hand—tests were excluded]) of digital active tests across the 3 studies were excluded from the analysis due to QC criteria not being met, indicating improper test execution (Multimedia Appendix 4). For the other tests, the QC fail rates were higher. The percentage of improperly executed Draw-A-Shape tests was higher among participants with manifest HD across the 3 studies in comparison with participants with premanifest HD and control participants in the Digital-HD study. Across the 3 studies, 8.60% (HD NHS: 94/1093) to 10% (OLE study: 33/331) and 13.05% (HD NHS: 140/1073) to 20.1% (OLE study: 66/329) of Draw-A-Shape tests performed by participants with manifest HD with the dominant (D) and nondominant (ND) hands, respectively, failed to pass the QC criteria. Moreover, an analysis of the per-subject QC pass rate for the Draw-A-Shape tests showed that this rate is negatively correlated with UHDRS-TMS and Maximal Chorea upper limb scores (Multimedia Appendix 5). Study participants were instructed to carry out the Walking, U-Turn, and Balance tests with the phone placed in the provided pouch around the waist. For the Walking test, it was found that not adhering to this instruction and carrying the device in the pocket instead resulted in skewed step frequency variance values. As a result, these test instances were discarded, which amounted to 17.5% (Digital-HD, premanifest HD: 40/228) to 30.9% (OLE study: 84/272) of all Walking tests. To be consistent, the same criterion was also applied to the Balance and the U-Turn tests, resulting in similar percentages of tests being discarded. It should be noted though that despite the relatively high number of discarded tests, only 11% (19/172), 12.8% (23/179), and 13.8% (25/181) of all participants were lost for subsequent analysis for the Walking, U-Turn, and Balance tests, respectively.

### Adherence

The active tests, excluding the Walking test, required on average (median) 8 to 9 minutes (OLE: 8:49 minutes, HD NHS: 8:59 minutes, and Digital-HD: 8:18 minutes) for the days without the SWR, the SDMT, and the EuroQol 5-dimension 5-level, and 14 to 15 minutes (OLE: 14:52 minutes and HD NHS: 14:18 minutes) for days with these nondaily tests. For Digital-HD, the nondaily tests were split over multiple days, leading to an average test time of 9 to 11 minutes for these days. In the OLE study, participants completed 1164 out of 1294 active tests (89.95%). Participants in the HD NHS and Digital-HD study performed a total of 2025 out of 2812 (72.01%) and 1454 out of 2108 (68.98%) tests, respectively.

### Test-Retest Reliability

Test-retest reliability was good to excellent for the active tests across the 3 studies and varied from 0.89 (95% CI 0.83-0.93) to 0.98 (95% CI 0.97-0.99; Table 3).

**Table 3:** Test-retest reliability intraclass correlation coefficients (ICCs) for the tests and selected face valid features.

Test	Clinical score	Digital test feature			Test-retest ICC of digital test				
			D/ND^a^ hand		OLE^b^ study		HD^c^ NHS^d^		Digital-HD
SDMT^e^	Number of correct answers for in-clinic SDMT	Number of correct answers (95% CI)	N/A^f^	0.94 (0.77-0.98)		0.93 (0.65-0.97)		0.98 (0.89-0.99)	
SWR^g^	Number of correctly read words for in-clinic SWR	Number of correctly read words (95% CI)	N/A	0.92 (0.85-0.95)		0.93 (0.90-0.96)		0.96 (0.94-0.98)	
Speeded Tapping	Mean intertap interval for in-clinic Speeded Tapping	Mean intertap interval (ms; 95% CI)	D^h^	0.94 (0.86-0.98)		0.97 (0.95-0.98)		0.98 (0.97-0.99)	
Speeded Tapping	Mean intertap interval for in-clinic Speeded Tapping	Mean intertap interval (ms; 95% CI)	ND^i^	0.96 (0.88-0.98)		0.97 (0.95-0.98)		0.97 (0.96-0.98)	
Draw-A-Shape	UHDRS^j^ Finger Taps	Spiral drawing speed variability (mm/s; 95% CI)	D	0.93 (0.85-0.97)		0.93 (0.87-0.96)		0.91 (0.85-0.95)	
Draw-A-Shape	UHDRS Finger Taps	Spiral drawing speed variability (mm/s; 95% CI)	ND	0.93 (0.84-0.97)		0.92 (0.87-0.95)		0.97 (0.94-0.98)	
Chorea	UHDRS Maximal Chorea upper limb	Sway path (m/s^2^; 95% CI)	D	0.96 (0.92-0.98)		0.96 (0.94-0.97)		0.98 (0.96-0.99)	
Chorea	UHDRS Maximal Chorea upper limb	Sway path (m/s^2^; 95% CI)	ND	0.97 (0.94-0.99)		0.94 (0.90-0.96)		0.98 (0.97-0.99)	
Balance	Balance score	Sway path (m/s^2^; 95% CI)	N/A	0.91 (0.73-0.97)		0.89 (0.83-0.93)		0.94 (0.89-0.97)	
U-Turn	TMS^k^	Median turn speed (rad/sec; 95% CI)	N/A	0.95 (0.89-0.98)		0.95 (0.92-0.97)		0.94 (0.91-0.97)	
Walking	TMS	Step frequency variance (Hz^2^; 95% CI)	N/A	0.95 (0.88-0.98)		0.93 (0.89-0.96)		0.95 (0.82-0.97)	

^a^D/ND: dominant/nondominant.

^b^OLE: open-label extension.

^c^HD: Huntington disease.

^d^NHS: Natural History Study.

^e^SDMT: Symbol Digit Modalities Test.

^f^N/A: not applicable.

^g^SWR: Stroop Word Reading.

^h^D: dominant.

^i^ND: nondominant.

^j^UHDRS: Unified HD Rating Scale.

^k^TMS: Total Motor Score.

### Clinical Cross-sectional Validity

#### Convergent Validity of Sensor-Based Measures With Standard Clinical Outcome Measures

**Figure 2:**
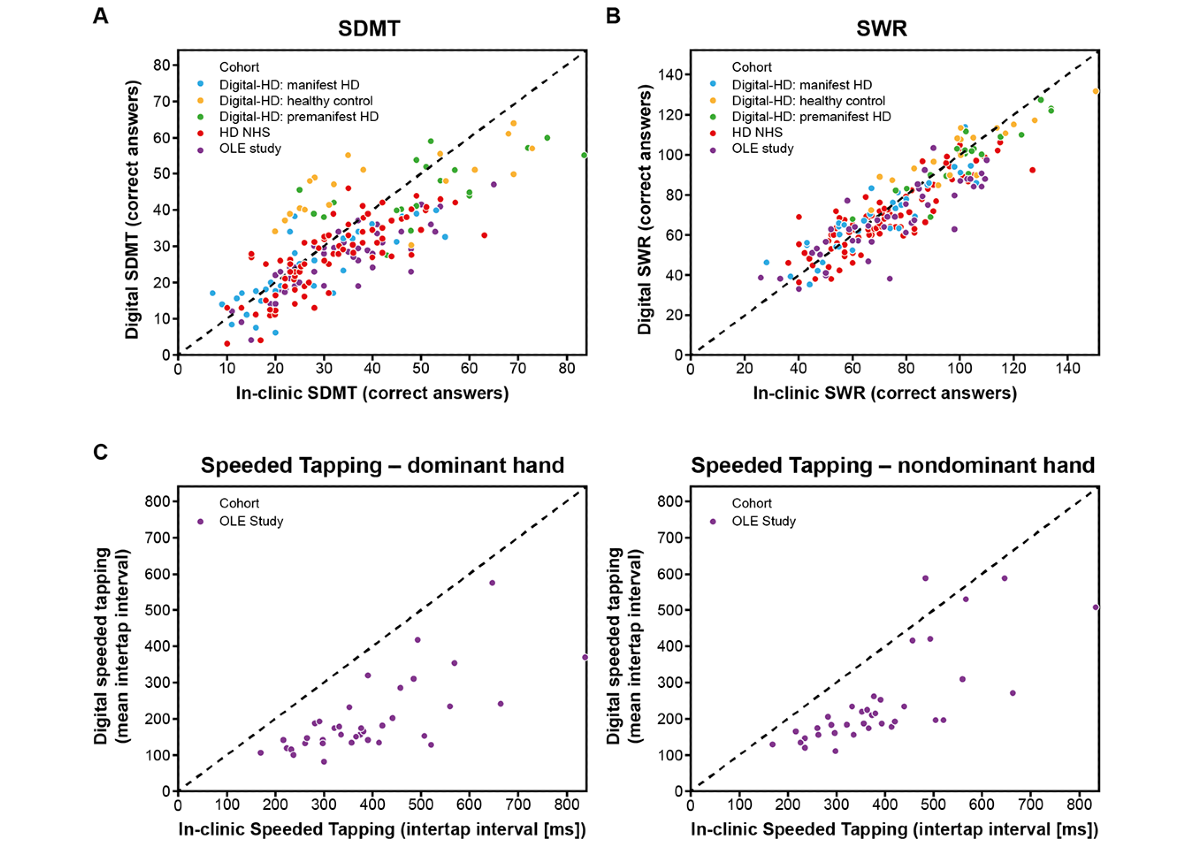
Clinical validity of digital cognitive tests and digital Speeded Tapping test. (A) Correlation of in-clinic Symbol Digit Modalities Test (SDMT) with digital SDMT. (B) Correlation of in-clinic Stroop Word Reading (SWR) test with digital SWR test. (C) Correlation of in-clinic Speeded Tapping test with digital Speeded Tapping test. HD: Huntington disease; NHS: Natural History Study; OLE: open-label extension.

Across the 3 studies for participants with manifest HD, the digital SDMT and SWR tests were strongly associated with the in-clinic SDMT (OLE: *r*=0.85, HD NHS: *r*=0.79, Digital-HD [manifest HD cohort]: *r*=0.80; *P*<.001 for all) and SWR (OLE: *r*=0.84, HD NHS: *r*=0.87, Digital-HD [manifest HD cohort]: 0.90; *P*<.001 for all tests), respectively (Figures 2A and 2B, Table 4, and Multimedia Appendix 6).

**Figure 3:**
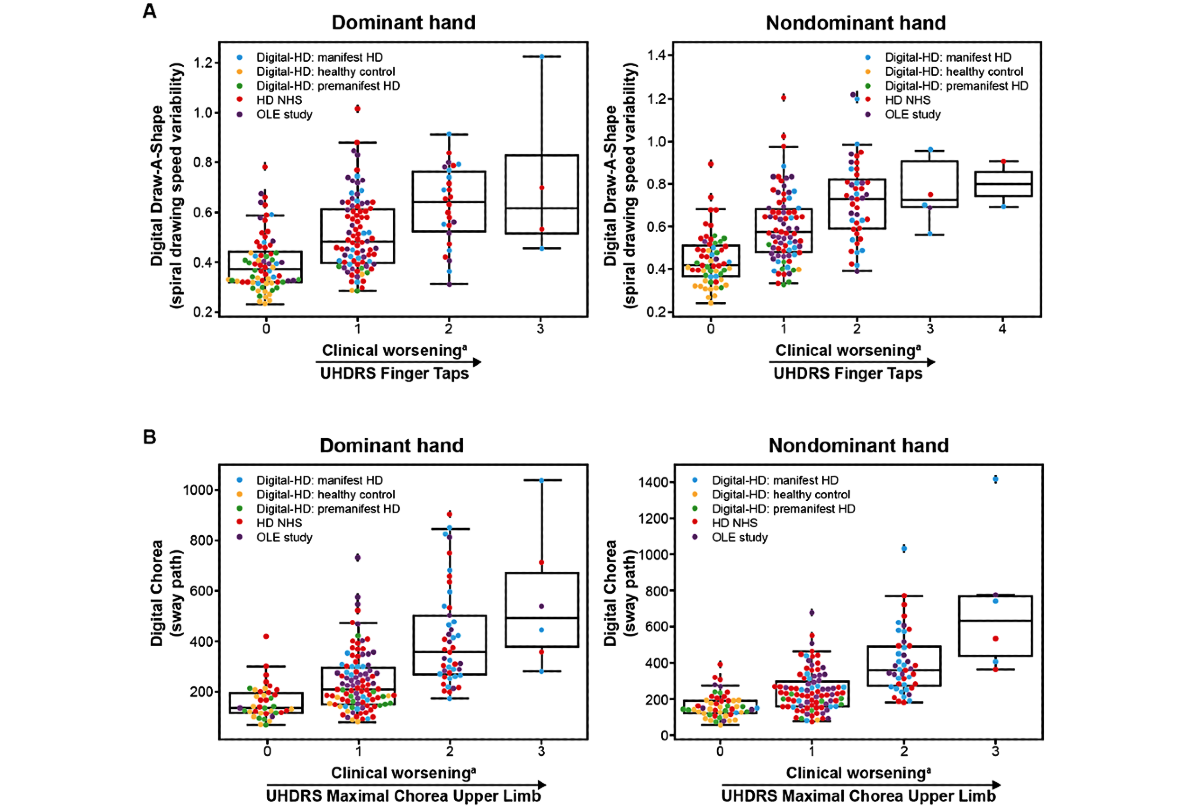
Clinical validity of Draw-A-Shape and digital Chorea tests. (A) Association of Unified HD Rating Scale (UHDRS) Finger Taps with spiral drawing speed variability during the Draw-A-Shape test. (B) Association of the UHDRS Maximal Chorea upper limb item with the log sway path measured during the digital Chorea test. ^a^Higher scores represent increased clinical worsening. HD: Huntington disease; NHS: Natural History Study; OLE: open-label extension.

For remote monitoring of upper body motor function, 3 active tests were used: Chorea test, Speeded Tapping test, and Draw-A-Shape test. In the OLE study, the digital Speeded Tapping test was strongly associated with the in-clinic Speeded Tapping test (D: *r*=0.70, ND: *r*=0.75; *P*<.001 for both; Figure 2C, Table 4, and Multimedia Appendix 6). The in-clinic Speeded Tapping test was not conducted in the HD NHS and the Digital-HD study. The spiral drawing speed variability showed moderate association with the UHDRS Finger Taps item across the 3 studies for participants with manifest HD when using the ND hand (OLE: *ρ*=0.47, *P*=.001; HD NHS: *ρ*=0.47, *P*<.001; Digital-HD [manifest HD cohort]: *ρ*=0.57, *P*<.001; Figure 3A, Table 4, and Multimedia Appendix 6). When using the D hand, the spiral drawing speed variability showed moderate association with the UHDRS Finger Taps item in the HD NHS (*ρ*=0.41; *P*<.001) and Digital-HD study (manifest HD cohort: *ρ*=0.55; *P*=.002). Sway path during Chorea tests showed moderate-to-strong associations across the studies, bilaterally with the UHDRS Maximal Chorea upper limb item (OLE: D/ND, *ρ*=0.50/0.58, HD NHS: D/ND, *ρ*=0.46/0.45, Digital-HD [manifest HD cohort]: D/ND, *ρ*=0.47/0.65; *P*<.001 for all except *P*=.006 for Digital-HD: D; Figure 3B, Table 4, and Multimedia Appendix 6).

**Figure 4:**
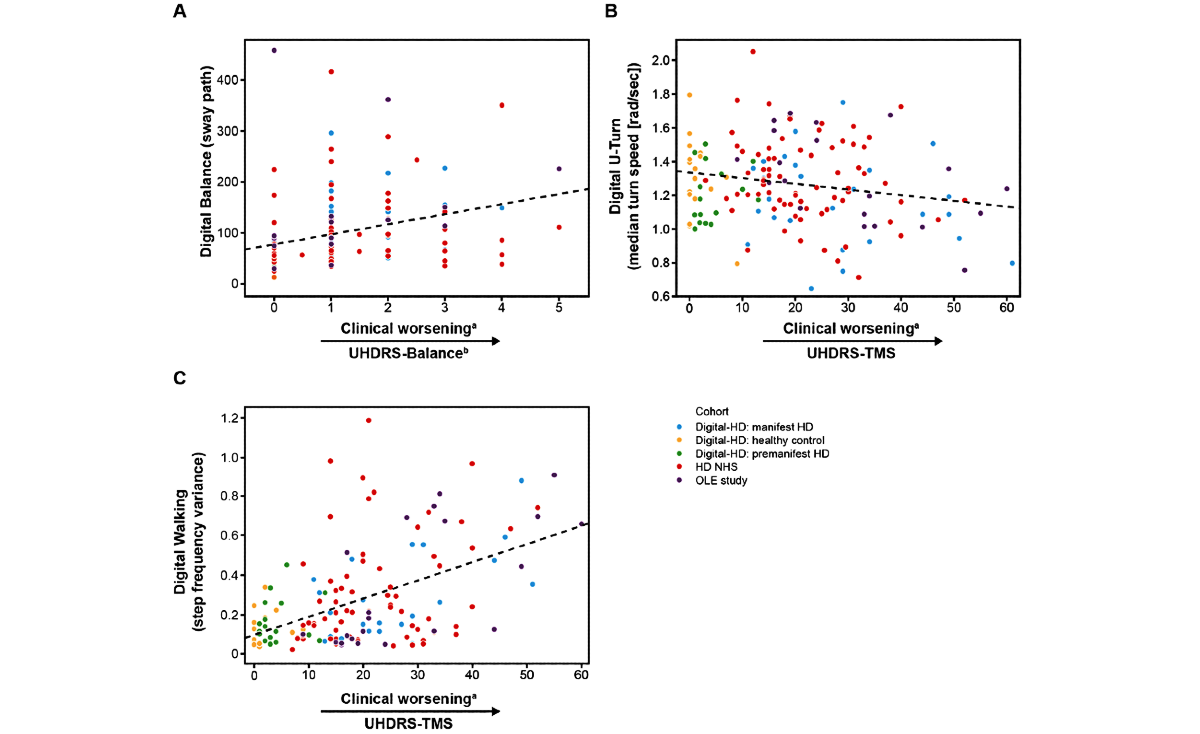
Clinical validity of digital whole body and lower limb tests. (A) Comparison of the balance score with the logarithm of the sway path based on smartphone signal while standing still. (B) Comparison of Unified HD Rating Scale-Total Motor Score (UHDRS-TMS) with the median turn speed during the U-Turn test. (C) Comparison of the UHDRS-TMS with the step frequency variance during the Walking test. The dotted line in (B) and (C) shows the regression line. ^a^Higher scores represent increased clinical worsening. ^b^Balance is the sum of UHDRS-TMS Retropulsion Pull test item and UHDRS-TMS Tandem Walking test item scores. HD: Huntington disease; NHS: Natural History Study; OLE: open-label extension.

For whole body and lower limb tests, significant weak-to-moderate associations were found with respective UHDRS items across the 3 studies for participants with manifest HD with the exception of median turn speed during the U-Turn test in the HD NHS and the Digital-HD study, and sway path during the Balance test in the Digital-HD study (Figure 4, Table 4, and Multimedia Appendix 6): sway path during the Balance test was associated with the in-clinic balance score (OLE: *ρ*=0.51, HD NHS: *ρ*=0.28, Digital-HD [manifest HD cohort]: *ρ*=0.24; *P*=.23), median turn speed when doing U-turns with UHDRS-TMS (OLE: *ρ*=–0.51, HD NHS: *ρ*=–0.16; *P*=.18, Digital-HD [manifest HD cohort]: *ρ*=–0.20; *P*=.32), and step frequency variance while walking for 2 minutes with UHDRS-TMS (OLE: *ρ*=0.71, HD NHS: *ρ*=0.26, and Digital-HD [manifest HD cohort]: *ρ*=0.47).

Using data from participants with premanifest HD in the Digital-HD study, the digital SDMT and SWR tests were strongly associated with the in-clinic SDMT (*r*=0.64; *P*=.002) and SWR (*r*=0.91; *P*<.001) tests, and sway path during the Chorea test showed moderate association with the UHDRS Chorea item when using the D hand (*r*=0.58; *P*=.01).

**Table 4:** Correlation coefficients between clinical scores and digital tests.

Test	Clinical score	Digital test feature			Correlation coefficient between clinical score and digital test				
					OLE^a^ study	HD^b^ NHS^c^	Digital-HD study		
				D/N^d^ hand			Healthy controls	Premanifest HD	Manifest HD
SDMT^e^	Number of correct answers for in-clinic SDMT	Number of correct answers (95% CI)	N/A^f^		0.85 (0.73 to 0.91)^g,h^	0.79 (0.69 to 0.86)^g,h^	0.68 (0.35 to 0.86)^g,h^	0.64 (0.28 to 0.84)^h,i^	0.80 (0.65 to 0.89)^g,h^
SWR^j^	Number of correctly read words for in-clinic SWR	Number of correctly read words (95% CI)	N/A		0.84 (0.72 to 0.91)^g,h^	0.87 (0.80 to 0.91) ^g,h^	0.87 (0.69 to 0.95) ^g,h^	0.91 (0.79 to 0.97)^g,h^	0.90 (0.82 to 0.95)^g,h^
Speeded Tapping	Mean intertap interval for in-clinic Speeded Tapping	Mean intertap interval (ms; 95% CI)		D^k^	0.70 (0.49 to 0.84)^g,h^	—^l^	—^l^	—^l^	—^l^
Speeded Tapping	Mean intertap interval for in-clinic Speeded Tapping	Mean intertap interval (ms; 95% CI)		ND^m^	0.75 (0.56 to 0.86)^g,h^	—^l^	—^l^	—^l^	—^l^
Draw-A-Shape	UHDRS^n^ Finger Taps	Spiral drawing speed variability (mm/s; 95% CI)		D	0.19 (−0.12 to 0.47)	0.41 (0.21 to 0.58)^g^	0.13 (−0.35 to 0.55)	0.02 (−0.44 to 0.47)	0.55 (0.23 to 0.76)^i^
Draw-A-Shape	UHDRS Finger Taps	Spiral drawing speed variability (mm/s; 95% CI)		ND	0.47 (0.20 to 0.68)^i^	0.47 (0.27 to 0.62)^g^	0.21 (−0.28 to 0.62)	−0.17 (−0.58 to 0.31)	0.57 (0.29 to 0.77)^g^
Chorea	UHDRS Maximal Chorea upper limb	Sway path (m/s^2^; 95% CI)		D	0.50 (0.23 to 0.70)^g^	0.46 (0.27 to 0.62)^g^	−0.06 (−0.50 to 0.41)	0.58 (0.17 to 0.82)^i^	0.47 (0.15 to 0.70)^i^
Chorea	UHDRS Maximal Chorea upper limb	Sway path (m/s^2^; 95% CI)		ND	0.58 (0.34 to 0.75)^g^	0.45 (0.25 to 0.61)^g^	−0.26 (−0.64 to 0.22)	0.27 (−0.22 to 0.66)	0.65 (0.40 to 0.81)^g^
Balance	Balance score	Sway path (m/s^2^; 95% CI)			0.51 (0.05 to 0.79)^o^	0.28 (0.05 to 0.48)^o^	−0.20 (−0.62 to 0.30)	—^p^	0.24 (−0.16 to 0.56)
U-Turn	TMS^q^	Median turn speed (rad/sec; 95% CI)			−0.51 (−0.77 to −0.09)^o^	−0.16 (−0.38 to −0.07)	−0.19 (−0.61 to 0.32)	−0.22 (−0.64 to 0.32)	−0.20 (−0.55 to 0.20)
Walking	TMS	Step frequency variance (Hz^2^; 95% CI)			0.71 (0.42 to 0.87)^g^	0.26 (0.02 to 0.47)^o^	0.32 (−0.21 to 0.70)	0.05 (−0.44 to 0.52)	0.47 (0.06 to 0.72)^o^

^a^OLE: open-label extension.

^b^HD: Huntington disease.

^c^NHS: Natural History Study.

^d^D/ND: dominant/nondominant.

^e^SDMT: Symbol Digit Modalities Test.

^f^N/A: not applicable.

^g^*P*<.001.

^h^Indicates Pearson correlation coefficient; Spearman correlation coefficients are used otherwise.

^i^*P*<.01.

^j^SWR: Stroop Word Reading.

^k^D: dominant.

^l^The in-clinic Speeded Tapping test was not conducted in the HD NHS and Digital-HD study.

^m^ND: nondominant.

^n^UHDRS: Unified HD Rating Scale.

^o^*P*<.05.

^p^Data not available.

^q^TMS: Total Motor Score.

#### Known-Groups Validity of Sensor-Based Measures

**Figure 5:**
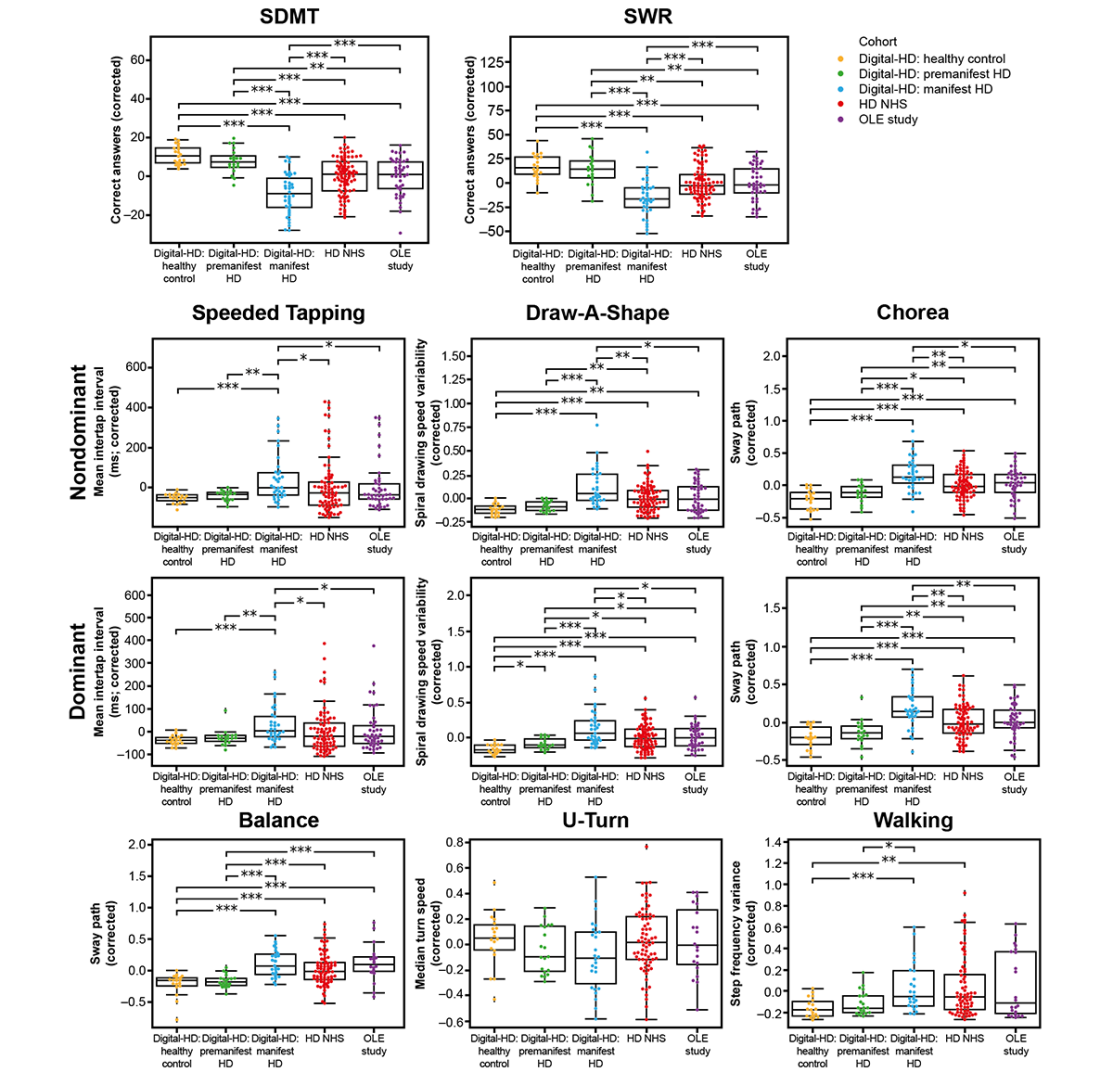
Known-groups validity of digital active tests. All 3 studies were used to compare sensor-derived feature values between the control, premanifest HD, and manifest HD groups to determine an association between digital feature value and disease status or stage. *P<.05; **P<.01; ***P<.001. HD: Huntington disease; NHS: Natural History Study; OLE: open-label extension; SDMT: Symbol Digit Modalities Test; SWR: Stroop Word Reading.

All 3 studies were used to determine the cross-sectional association of each of the 6 motor features and 2 cognitive features to disease status (premanifest HD vs manifest HD vs controls). The results demonstrated that, across features, there was an increasing pattern of abnormality as a function of disease stage (controls vs manifest HD and premanifest HD vs manifest HD), indicating that the digital motor and cognitive features are disease-status associated (Figure 5 and Multimedia Appendix 7). The results within disease stage from the Digital-HD study (manifest HD cohort), OLE study, and HD NHS showed a consistent level of abnormality across the features assessed in the Balance, U-Turn, and Walking tests.

## Discussion

### Principal Findings

Continuous remote digital assessment of motor and cognitive features of HD appears feasible, reliable, and valid in cross-section across 3 independent HD cohorts. The selected features are robustly associated with disease stage, as well as clinical severity, as measured by standard in-clinic assessments. Overall adherence to the active tests was good to excellent across the 3 studies, demonstrating that the length and number of daily tests were acceptable to most participants.

### Interpretation and Comparison With Prior Work

Both cognitive and motor features, except for the turn speed during the U-Turn test in the HD NHS and Digital-HD study, sway path during the Balance test in the Digital-HD study, and spiral drawing speed variability during the Draw-A-Shape test in the OLE study, were significantly correlated with corresponding in-clinic assessments. Good overall convergent validity suggests that the tests measure the same domains as the in-clinic counterparts. Of all digital tests, the cognitive tests demonstrated the strongest correlations with analogous in-clinic tests. Importantly, both digital and standard cognitive outcomes studied here are based upon the same underlying pseudocontinuous scale, which may explain the high degree of association observed. Indeed, a prior and much smaller study of 4 smartphone-based cognitive assessments in participants with HD showed varying degrees of association between the digital cognitive measures and Enroll-HD cognitive tasks, which included SWR and SDMT (Pearson correlation coefficients 0.36-0.68) [12]. Overall, 1 of the 4 digital cognitive assessments showed no significant correlations with Enroll-HD cognitive tasks [12], indicating that this digital assessment may not be measuring the same constructs that the Enroll-HD cognitive tasks are measuring.

In this study, digital motor tests, though analogous to the in-clinic motor assessment in the UHDRS, are quantitative measures that may offer advantages for improved objectivity and sensitivity, whereas the UHDRS scores are based on Likert-type clinician-rated scales. Notably, sway path during digital Chorea tests showed moderate-to-strong associations across the studies, bilaterally with the UHDRS Maximal Chorea upper limb item (Spearman correlation coefficients 0.45-0.65). These results are consistent with a pilot study of a smartphone app for HD that showed a positive correlation between a digital assessment of chorea and the clinical UHDRS Maximal Chorea score (*r*=0.53 for the left hand and *r*=0.54 for the right hand), although the effect was not found to be statistically significant in this small study of 8 participants with manifest HD [11].

Both cognitive features and 2 of the 6 motor features, Speeded Tapping and Chorea, had a low proportion of improperly executed tests (eg, ≤8%). The relatively high amount of improperly executed, and therefore nonevaluable, tests for the Draw-A-Shape task could be owing to how the test was implemented. The attempt to draw a shape is considered as completed as soon as the participant lifts a finger from the screen. This implementation detail could explain why participants with manifest HD had a higher proportion of improperly executed tests. Further research may indicate if changing the implementation to be more tolerant to lifting the finger from the screen will result in a higher proportion of correctly executed tests. The high proportion of failed tests observed with the Balance, U-Turn, and Walking tests was due to the exclusion of data that were collected from participants who performed the tests with the smartphone in their trouser pocket instead of the provided running belt; these data were excluded to account for any possible influence of sensor placement differences on the digital measures.

All sensor-based features had excellent test-retest reliability (ICCs≥0.8). Generally low QC failure, high reliability, and good adherence indicate that these measures possess the properties required to be used as outcome measures in clinical trials. The difference in adherence overall between studies is likely due to the difference in study design, where participants in an interventional trial, in this case the OLE study, are more likely to be motivated compared with participants in observational trials, as with the HD NHS and Digital-HD study. Furthermore, although adherence to the active tests was acceptable across the 3 studies over the 4-week study period, the digital monitoring platform should be evaluated over a longer period to further assess feasibility.

Of note, the digital Speeded Tapping test showed a shift toward shorter mean intertap intervals relative to the in-clinic analog, a shift that most likely reflects a systematic difference between the different devices and platforms used. In some cases, the selected digital features (eg, speed variability of the Draw-A-Shape test) could not map directly to the in-clinic analog (eg, UHDRS Finger Taps item), which may in part explain the lower degree of association between the measures. However, novel assessment of fine motor skills has the potential to detect small changes in motor function that may not otherwise be detected by traditional in-clinic assessments, as supported by the ability of the Draw-A-Shape test to differentiate participants with premanifest HD from controls.

### Strengths and Limitations

In summary, data from remote patient-driven digital monitoring systems have the potential to advance insights into HD disease features and progression that may enable improved clinical trial design and disease management. As demonstrated in this study, the Roche HD Digital Monitoring Platform appears to fulfill the criteria of cross-sectional validation required for a novel platform to be useful in this context. An important limitation of this study is the cross-sectional nature of the data and the lack of a comprehensive evaluation of the platform’s clinical validity. Accordingly, the next goal for the platform is to demonstrate sensitivity to clinical change over time and ability to measure drug effect. Such additional longitudinal data are critical to judge the true value of the digital approach versus the standard approach, and these data are presently being generated across the Roche tominersen clinical development program in interventional and observational contexts. Another limitation is the limited understanding of how these prespecified features are linked to what matters for patients in daily life. As explained above, the feature preselection was driven by literature and expert input and as such is mainly signal identification driven. Following recommendations previously outlined for the development of meaningful digital measures [41,42], a qualitative research study to investigate what matters most for patients in their daily life in relation to the HD Digital Monitoring Platform is ongoing. One core strength of digital testing is that it entails the high-frequency collection of data. This enables the development of a broader feature space that has the potential to show even stronger signals, such as features that can differentiate between healthy controls and those with premanifest HD (as demonstrated in this study) and show greater relevance to what matters to patients.

### Conclusions

Taken together, the analyses presented support the use of wearable devices and mobile apps to provide further insight into HD disease features and clinical progression previously not possible with standard clinical assessments, enabling improved clinical trial design and, potentially, disease management.

## Appendix

Multimedia Appendix 1 Animated video describing the Roche Huntington disease digital monitoring platform.

Multimedia Appendix 2 Screenshots of active tests on the Roche HD Monitoring app.

Multimedia Appendix 3 Quality control pass criteria for digital active tests.

Multimedia Appendix 4 Quality control summary statistics.

Multimedia Appendix 5 Correlation statistics for the QC pass rate for the Draw-A-Shape test with UHDRS-TMS and Maximal Chorea upper limb item.

Multimedia Appendix 6 Correlation coefficients and *P* values between clinical score and digital test.

Multimedia Appendix 7 *P* values for known-groups validity of digital active tests.

## Abbreviations

CAG: cytosine adenine guanine
D: dominant
HD: Huntington disease
ICC: intraclass correlation coefficient
IRB: Institutional Review Board
ND: nondominant
NHS: Natural History Study
OLE: open-label extension
QC: quality control
SDMT: Symbol Digit Modalities Test
SWR: Stroop Word Reading
TFC: Total Functional Capacity
TMS: Total Motor Score
UHDRS: Unified HD Rating Scale

## Data Availability

For eligible studies, qualified researchers may request access to individual patient-level clinical data through a data request platform. At the time of writing, this request platform is Vivli [43]. Up-to-date details on Roche's global policy on the sharing of clinical information and how to request access to related clinical study documents are available on the Roche website [44]. Anonymised records for individual patients across more than one data source external to Roche can not, and should not, be linked due to a potential increase in risk of patient reidentification.

## References

[1] Bates GP, Dorsey R, Gusella JF, Hayden MR, Kay C, Leavitt CR, Nance M, Ross CA, Scahill RI, Wetzel R, Wild EJ, Tabrizi SJ (2015). Huntington disease. Nat Rev Dis Primers.

[2] Roos RA (2010). Huntington's disease: a clinical review. Orphanet J Rare Dis.

[3] Dorsey ER, Papapetropoulos S, Xiong M, Kieburtz K (2017). The first frontier: digital biomarkers for neurodegenerative disorders. Digit Biomark.

[4] Post B, Merkus MP, de Bie RM, de Haan RJ, Speelman JD (2005). Unified Parkinson's disease rating scale motor examination: are ratings of nurses, residents in neurology, and movement disorders specialists interchangeable?. Mov Disord.

[5] Teipel S, König A, Hoey J, Kaye J, Krüger F, Robillard JM, Kirste T, Babiloni C (2018). Use of nonintrusive sensor-based information and communication technology for real-world evidence for clinical trials in dementia. Alzheimers Dement.

[6] Andrzejewski KL, Dowling AV, Stamler D, Felong TJ, Harris DA, Wong C, Cai H, Reilmann R, Little MA, Gwin JT, Biglan KM, Dorsey ER (2016). Wearable sensors in Huntington disease: a pilot study. J Huntingtons Dis.

[7] Adams JL, Dinesh K, Xiong M, Tarolli CG, Sharma S, Sheth N, Aranyosi AJ, Zhu W, Goldenthal S, Biglan KM, Dorsey ER, Sharma G (2017). Multiple wearable sensors in Parkinson and Huntington disease individuals: a pilot study in clinic and at home. Digit Biomark.

[8] Cohen S, Waks Z, Elm JJ, Gordon MF, Grachev ID, Navon-Perry L, Fine S, Grossman I, Papapetropoulos S, Savola J (2018). Characterizing patient compliance over six months in remote digital trials of Parkinson's and Huntington disease. BMC Med Inform Decis Mak.

[9] Dinesh K, Snyder CW, Xiong M, Tarolli CG, Sharma S, Dorsey ER, Sharma G, Adams JL (2020). A longitudinal wearable sensor study in Huntington's disease. J Huntingtons Dis.

[10] Tortelli R, Rodrigues FB, Wild EJ (2021). The use of wearable/portable digital sensors in Huntington's disease: a systematic review. Parkinsonism Relat Disord.

[11] Waddell EM, Dinesh K, Spear KL, Elson MJ, Wagner E, Curtis MJ, Mitten DJ, Tarolli CG, Sharma G, Dorsey ER, Adams JL (2021). GEORGE®: a pilot study of a smartphone application for Huntington's disease. J Huntingtons Dis.

[12] McLaren B, Andrews SC, Glikmann-Johnston Y, Mercieca EC, Murray NW, Loy C, Bellgrove MA, Stout JC (2021). Feasibility and initial validation of 'HD-Mobile', a smartphone application for remote self-administration of performance-based cognitive measures in Huntington's disease. J Neurol.

[13] Kassavetis P, Saifee TA, Roussos G, Drougkas L, Kojovic M, Rothwell JC, Edwards MJ, Bhatia KP (2015). Developing a tool for remote digital assessment of Parkinson's disease. Mov Disord Clin Pract.

[14] Lipsmeier F, Taylor KI, Kilchenmann T, Wolf D, Scotland A, Schjodt-Eriksen J, Cheng WY, Fernandez-Garcia I, Siebourg-Polster J, Jin L, Soto J, Verselis L, Boess F, Koller M, Grundman M, Monsch AU, Postuma RB, Ghosh A, Kremer T, Czech C, Gossens C, Lindemann M (2018). Evaluation of smartphone-based testing to generate exploratory outcome measures in a phase 1 Parkinson's disease clinical trial. Mov Disord.

[15] Arora S, Venkataraman V, Zhan A, Donohue S, Biglan KM, Dorsey ER, Little MA (2015). Detecting and monitoring the symptoms of Parkinson's disease using smartphones: a pilot study. Parkinsonism Relat Disord.

[16] Lee CY, Kang SJ, Hong SK, Ma HI, Lee U, Kim YJ (2016). A validation study of a smartphone-based finger tapping application for quantitative assessment of bradykinesia in Parkinson's disease. PLoS One.

[17] Prince J, Arora S, de Vos M (2018). Big data in Parkinson's disease: using smartphones to remotely detect longitudinal disease phenotypes. Physiol Meas.

[18] Elm JJ, Daeschler M, Bataille L, Schneider R, Amara A, Espay AJ, Afek M, Admati C, Teklehaimanot A, Simuni T (2019). Feasibility and utility of a clinician dashboard from wearable and mobile application Parkinson's disease data. NPJ Digit Med.

[19] Printy BP, Renken LM, Herrmann JP, Lee I, Johnson B, Knight E, Varga G, Whitmer D (2014). Smartphone application for classification of motor impairment severity in Parkinson's disease. Annu Int Conf IEEE Eng Med Biol Soc.

[20] Zhan A, Mohan S, Tarolli C, Schneider RB, Adams JL, Sharma S, Elson MJ, Spear KL, Glidden AM, Little MA, Terzis A, Dorsey ER, Saria S (2018). Using smartphones and machine learning to quantify Parkinson disease severity: the mobile Parkinson disease score. JAMA Neurol.

[21] Isaacson SH, Boroojerdi B, Waln O, McGraw M, Kreitzman DL, Klos K, Revilla FJ, Heldman D, Phillips M, Terricabras D, Markowitz M, Woltering F, Carson S, Truong D (2019). Effect of using a wearable device on clinical decision-making and motor symptoms in patients with Parkinson's disease starting transdermal rotigotine patch: a pilot study. Parkinsonism Relat Disord.

[22] Vuong K, Canning CG, Menant JC, Loy CT (2018). Gait, balance, and falls in Huntington disease. Handb Clin Neurol.

[23] Hamilton JM, Salmon DP, Corey-Bloom J, Gamst A, Paulsen JS, Jerkins S, Jacobson MW, Peavy G (2003). Behavioural abnormalities contribute to functional decline in Huntington's disease. J Neurol Neurosurg Psychiatry.

[24] Nehl C, Paulsen JS, Huntington Study Group (2004). Cognitive and psychiatric aspects of Huntington disease contribute to functional capacity. J Nerv Ment Dis.

[25] Ready RE, Mathews M, Leserman A, Paulsen JS (2008). Patient and caregiver quality of life in Huntington's disease. Mov Disord.

[26] Simpson JA, Lovecky D, Kogan J, Vetter LA, Yohrling GJ (2016). Survey of the Huntington's disease patient and caregiver community reveals most impactful symptoms and treatment needs. J Huntingtons Dis.

[27] Lipsmeier F, Cheng W, Wolf D, Zhang YP, Kilchenmann T, Bamdadian A, Smith A, Wild E, Schobel S, Czech C, Gossens C, Lindermann M (2018). Digital, high-frequency, long-term monitoring of motor and non-motor symptoms in huntington’s disease (hd) patients. J Neurol Neurosurg Psychiatry.

[28] Lipsmeier F, Simillion C, Bamdadian A, Smith A, Schobel S, Czech C, Gossens C, Weydt P, Wild E, Lindermann M (2019). Preliminary reliability and validity of a novel digital biomarker smartphone application to assess cognitive and motor symptoms in Huntington’s disease (HD) (P1.8-042). Neurology.

[29] Kremer H P H, Hungtington Study Group (1996). Unified Huntington's Disease Rating Scale: reliability and consistency. Huntington Study Group. Mov Disord.

[30] Stroop JR (1935). Studies of interference in serial verbal reactions. J Exp Psychol.

[31] Smith A (1973). Symbol Digit Modalities Test.

[32] Michell AW, Goodman AO, Silva AH, Lazic SE, Morton AJ, Barker RA (2008). Hand tapping: a simple, reproducible, objective marker of motor dysfunction in Huntington's disease. J Neurol.

[33] Salomonczyk D, Panzera R, Pirogovosky E, Goldstein J, Corey-Bloom J, Simmons R, Gilbert PE (2010). Impaired postural stability as a marker of premanifest Huntington's disease. Mov Disord.

[34] Tian JR, Herdman SJ, Zee DS, Folstein SE (1991). Postural control in Huntington's disease (HD). Acta Otolaryngol Suppl.

[35] Hausdorff JM, Cudkowicz ME, Firtion R, Wei JY, Goldberger AL (1998). Gait variability and basal ganglia disorders: stride-to-stride variations of gait cycle timing in Parkinson's disease and Huntington's disease. Mov Disord.

[36] Mancini M, Salarian A, Carlson-Kuhta P, Zampieri C, King L, Chiari L, Horak FB (2012). ISway: a sensitive, valid and reliable measure of postural control. J Neuroeng Rehabil.

[37] Cheng WY, Bourke AK, Lipsmeier F, Bernasconi C, Belachew S, Gossens C, Graves JS, Montalban X, Lindemann M (2021). U-turn speed is a valid and reliable smartphone-based measure of multiple sclerosis-related gait and balance impairment. Gait Posture.

[38] Moon Y, Sung J, An R, Hernandez ME, Sosnoff JJ (2016). Gait variability in people with neurological disorders: a systematic review and meta-analysis. Hum Mov Sci.

[39] Bechtel N, Scahill RI, Rosas HD, Acharya T, van den Bogaard SJ, Jauffret C, Say MJ, Sturrock A, Johnson H, Onorato CE, Salat DH, Durr A, Leavitt BR, Roos RA, Landwehrmeyer GB, Langbehn DR, Stout JC, Tabrizi SJ, Reilmann R (2010). Tapping linked to function and structure in premanifest and symptomatic Huntington disease. Neurology.

[40] Shrout PE, Fleiss JL (1979). Intraclass correlations: uses in assessing rater reliability. Psychol Bull.

[41] Taylor KI, Staunton H, Lipsmeier F, Nobbs D, Lindemann M (2020). Outcome measures based on digital health technology sensor data: data- and patient-centric approaches. NPJ Digit Med.

[42] Manta C, Patrick-Lake B, Goldsack JC (2020). Digital measures that matter to patients: a framework to guide the selection and development of digital measures of health. Digit Biomark.

[43] Our members. Vivli.

[44] Our commitment to data sharing. Roche.

