# Supplementary material for: A Remote Digital Monitoring Platform to Assess Cognitive and Motor Symptoms in Huntington Disease: Cross-sectional Validation Study
Source: J Med Internet Res. 2022 Jun 28;24(6):e32997. doi: 10.2196/32997 (PMC9277525; doi:10.2196/32997)
Supplement: Multimedia Appendix 2 [file jmir_v24i6e32997_app2.docx]

This is a Multimedia Appendix to a full manuscript published in the J Med Internet Res. For full copyright and citation information see [http://dx.doi.org/10.2196/jmir.32997](http://dx.doi.org/10.2196/jmir.xxxx)

Multimedia Appendix 2. Screenshots of active tests on the Roche HD mobile application

**
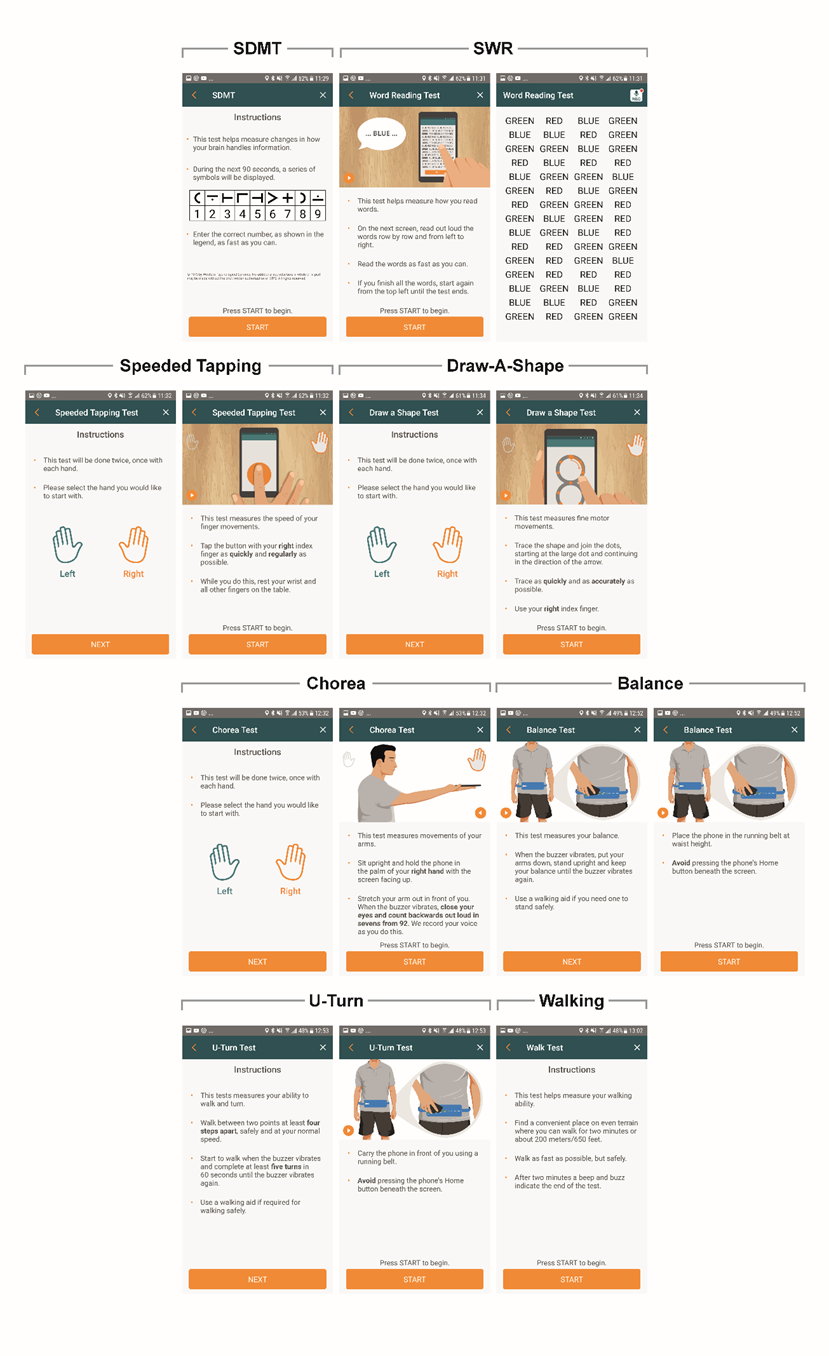
**
