# Supplementary material for: A Remote Digital Monitoring Platform to Assess Cognitive and Motor Symptoms in Huntington Disease: Cross-sectional Validation Study
Source: J Med Internet Res. 2022 Jun 28;24(6):e32997. doi: 10.2196/32997 (PMC9277525; doi:10.2196/32997)
Supplement: Multimedia Appendix 3 [file jmir_v24i6e32997_app3.docx]

This is a Multimedia Appendix to a full manuscript published in the J Med Internet Res. For full copyright and citation information see <http://dx.doi.org/10.2196/jmir.32997>

Multimedia Appendix 3. QC pass criteria for digital active tests.

| **Digital test** | **QC pass criteria** | **Implementation** |
| --- | --- | --- |
| SDMT | User does not skip through the test by giving arbitrary answers | At least 20% correct answers or mean time between answers more than 1.1 seconds |
| SWR | A minimum number of words can be detected from the voice recording | A least 10 voicing segments detected |
| Speeded Tapping | User does not use more than one finger to complete test | Fraction of multi-touch events is less than 0.05 |
| Draw-A-Shape | Shape (spiral) is correctly drawn | Drawn shape length is at least 0.5times reference shape length and at most 1.5 times. |
| Chorea | Phone is not placed on the table | Z-axis acceleration mean is less than 99.8% of total acceleration magnitude or standard deviation is more than 0.005 |
| Balance | Phone is not placed on the table and test is carried out with device in the pouch | Z-axis acceleration mean is less than 99.8% of total acceleration magnitude or standard deviation is more than 0.005; device is mainly in landscape orientation |
| U-Turn | User performs U-Turns and device is carried in the pouch | At least one U-Turn detected; device is mainly in landscape orientation |
| Walking | User walks for sufficiently long amount of time and device is carried in the pouch | At least 1 minute of gait and 50 steps detected; device is mainly in landscape orientation |

SDMT, Symbol Digit Modalities Test; SWR, Stroop Word Reading; QC, quality control.
