# Supplementary material for: A Remote Digital Monitoring Platform to Assess Cognitive and Motor Symptoms in Huntington Disease: Cross-sectional Validation Study
Source: J Med Internet Res. 2022 Jun 28;24(6):e32997. doi: 10.2196/32997 (PMC9277525; doi:10.2196/32997)
Supplement: Multimedia Appendix 4 [file jmir_v24i6e32997_app4.docx]

This is a Multimedia Appendix to a full manuscript published in the J Med Internet Res. For full copyright and citation information see [http://dx.doi.org/10.2196/jmir.32997](http://dx.doi.org/10.2196/jmir.xxxx)

Multimedia Appendix 4. QC summary statistics.

D, dominant; HD, Huntington’s disease; ND, non-dominant; NHS, Natural History Study; OLE, open-label extension; SDMT, Symbol Digit Modalities Test; SWR, Stroop Word Reading.

| **Digital test** | **Digital-HD study –**  **healthy control** | | | | **Digital-HD study –**  **premanifest HD** | | | | | **Digital-HD study –**  **manifest HD** | | | | **HD NHS** | | | | **OLE study** | | | |
| --- | --- | --- | --- | --- | --- | --- | --- | --- | --- | --- | --- | --- | --- | --- | --- | --- | --- | --- | --- | --- | --- |
|  | **Failed (%)** | **Failed (n)** | **Passed (n)** | **Total (N)** | **Failed (%)** | **Failed (n)** | **Passed (n)** | **Total (N)** | **Failed (%)** | | **Failed (n)** | **Passed (n)** | **Total**  **(N)** | **Failed (%)** | **Failed (n)** | **Passed (n)** | **Total (N)** | **Failed (%)** | **Failed (n)** | **Passed (n)** | **Total (N)** |
| SDMT | 0.00 | 0 | 73 | 73 | 0.00 | 0 | 81 | 81 | 0.00 | | 0 | 122 | 122 | 0.00 | 0 | 252 | 252 | 0.00 | 0 | 86 | 86 |
| SWR | 0·00 | 0 | 66 | 66 | 0·00 | 0 | 67 | 67 | 0·00 | | 0 | 108 | 108 | 0·00 | 0 | 241 | 241 | 0·00 | 0 | 131 | 131 |
| Speeded Tapping (D) | 0.43 | 1 | 231 | 232 | 0.76 | 2 | 261 | 263 | 5.26 | | 22 | 396 | 418 | 1.18 | 11 | 923 | 934 | 0.30 | 1 | 335 | 336 |
| Speeded Tapping (ND) | 0.41 | 1 | 242 | 243 | 0.38 | 1 | 263 | 264 | 1.27 | | 5 | 388 | 393 | 1.03 | 11 | 1054 | 1065 | 1.80 | 6 | 328 | 334 |
| Draw-A-Shape (D) | 0.44 | 1 | 224 | 225 | 1.66 | 4 | 237 | 241 | 9.81 | | 37 | 340 | 377 | 8.60 | 94 | 999 | 1093 | 9.97 | 33 | 298 | 331 |
| Draw-A-Shape (ND) | 2.15 | 5 | 228 | 233 | 0.79 | 2 | 250 | 252 | 14.04 | | 50 | 306 | 356 | 13.05 | 140 | 933 | 1073 | 20.06 | 66 | 263 | 329 |
| Chorea (D) | 4.85 | 11 | 216 | 227 | 3.94 | 10 | 244 | 254 | 1.23 | | 5 | 402 | 407 | 2.45 | 27 | 1075 | 1102 | 0.92 | 3 | 323 | 326 |
| Chorea (ND) | 6.72 | 16 | 222 | 238 | 4.96 | 13 | 249 | 262 | 1.55 | | 6 | 381 | 387 | 3.46 | 38 | 1060 | 1098 | 1.23 | 4 | 322 | 326 |
| Balance | 16.10 | 38 | 198 | 236 | 11.65 | 29 | 220 | 249 | 20.55 | | 82 | 317 | 399 | 29.80 | 320 | 754 | 1074 | 39.74 | 122 | 185 | 307 |
| U-Turn | 13.22 | 30 | 197 | 227 | 8.04 | 18 | 206 | 224 | 15.73 | | 59 | 316 | 375 | 27.13 | 283 | 760 | 1043 | 32.99 | 96 | 195 | 291 |
| Walking | 18.35 | 40 | 178 | 218 | 17.54 | 40 | 188 | 228 | 24.61 | | 79 | 242 | 321 | 26.06 | 240 | 681 | 921 | 30.88 | 84 | 188 | 272 |
