# Supplementary material for: A Remote Digital Monitoring Platform to Assess Cognitive and Motor Symptoms in Huntington Disease: Cross-sectional Validation Study
Source: J Med Internet Res. 2022 Jun 28;24(6):e32997. doi: 10.2196/32997 (PMC9277525; doi:10.2196/32997)
Supplement: Multimedia Appendix 5 [file jmir_v24i6e32997_app5.docx]

This is a Multimedia Appendix to a full manuscript published in the J Med Internet Res. For full copyright and citation information see [http://dx.doi.org/10.2196/jmir.32997](http://dx.doi.org/10.2196/jmir.xxxx)

Multimedia Appendix 5. Correlation statistics for the QC pass rate for the Draw-A-Shape test with UHDRS-TMS and Maximal Chorea upper limb item.

| **Handedness** | **Clinical score** | **Cohort** | r | p | n |
| --- | --- | --- | --- | --- | --- |
| Dominant | Maximal Chorea upper limb dominant | Digital-HD: healthy control | –0.07 | 0.764 | 20 |
|  |  | Digital-HD: premanifest HD | –0.21 | 0.365 | 20 |
|  |  | Digital-HD: manifest HD | –0.53 | 0.001 | 34 |
|  |  | HD NHS | –0.32 | 0.003 | 85 |
|  |  | OLE study | –0.32 | 0.034 | 43 |
|  | TMS | Digital-HD: healthy control | –0.21 | 0.377 | 20 |
|  |  | Digital-HD: premanifest HD | –0.33 | 0.152 | 20 |
|  |  | Digital-HD: manifest HD | –0.67 | 0.000 | 34 |
|  |  | HD NHS | –0.33 | 0.002 | 85 |
|  |  | OLE study | –0.40 | 0.007 | 43 |
| Non-dominant | Maximal Chorea upper limb  non-dominant | Digital-HD: healthy control | –0.14 | 0.544 | 20 |
|  |  | Digital-HD: premanifest HD | –0.40 | 0.080 | 20 |
|  |  | Digital-HD: manifest HD | –0.29 | 0.081 | 36 |
|  |  | HD NHS | –0.36 | 0.001 | 85 |
|  |  | OLE study | –0.36 | 0.019 | 43 |
|  | TMS | Digital-HD: healthy control | –0.32 | 0.164 | 20 |
|  |  | Digital-HD: premanifest HD | –0.37 | 0.110 | 20 |
|  |  | Digital-HD: manifest HD | –0.38 | 0.022 | 36 |
|  |  | HD NHS | –0.48 | 0.000 | 85 |
|  |  | OLE study | –0.45 | 0.003 | 43 |

HD, Huntington’s disease; NHS, Natural History Study; OLE, open-label extension; TMS, Total Motor Score.
