# Supplementary material for: A Remote Digital Monitoring Platform to Assess Cognitive and Motor Symptoms in Huntington Disease: Cross-sectional Validation Study
Source: J Med Internet Res. 2022 Jun 28;24(6):e32997. doi: 10.2196/32997 (PMC9277525; doi:10.2196/32997)
Supplement: Multimedia Appendix 6 [file jmir_v24i6e32997_app6.docx]

This is a Multimedia Appendix to a full manuscript published in the J Med Internet Res. For full copyright and citation information see <http://dx.doi.org/10.2196/jmir.32997>

Multimedia Appendix 6. Correlation coefficients and P values between clinical score and digital test.

| **Test** | **Clinical score** | **Digital test feature** | | **OLE study** | | **HD NHS** | | **Digital-HD study** | | | | | | |
| --- | --- | --- | --- | --- | --- | --- | --- | --- | --- | --- | --- | --- | --- | --- |
|  |  |  |  |  |  |  |  | **Healthy controls** | | **Premanifest HD** | | **Manifest HD** | | |
|  |  |  |  | **Coefficient (95% CI)** | ***P* value** | **Coefficient  (95% CI)** | ***P* value** | **Coefficient (95% CI)** | ***P* value** | **Coefficient  (95% CI)** | ***P* value** | **Coefficient (95% CI)** | ***P* value** |  |
| SDMT | Number of correct answers for in-clinic SDMT | Number of correct answers | | 0.85 (0.73–0.91)^a^ | *P*<.001 | 0.79 (0.69–0.86)^a^ | *P*<.001 | 0.68 (0.35–0.86)^a^ | *P*<.001 | 0.64 (0.28–0.84)^a^ | *P*=.002 | 0.80 (0.65–0.89)^a^ | *P*<.001 |  |
| SWR | Number of correctly read words for in-clinic SWR | Number of correctly read words | | 0.84 (0.72–0.91)^a^ | *P*<.001 | 0.87 (0.80–0.91)^a^ | *P*<.001 | 0.87 (0.69–0.95)^a^ | *P*<.001 | 0.91 (0.79–0.97)^a^ | *P*<.001 | 0.90 (0.82–0.95)^a^ | *P*<.001 |  |
| Speeded Tapping^b^ | Mean inter-tap interval for in-clinic Speeded Tapping | Mean inter-tap interval (ms) | D | 0.70 (0.49–0.84)^a^ | *P*<.001 | – | – | – | – | – | – | – | – |  |
|  |  |  | ND | 0.75 (0.56–0.86)^a^ | *P*<.001 | – | – | – | – | – | – | – | – |  |
| Draw-A-Shape | UHDRS Finger Taps | Spiral drawing speed variability (mm/s) | D | 0.19 (−0.12–0.47) | *P*=.23 | 0.41 (0.21–0.58) | *P*<.001 | 0.13 (−0.35 to 0.55) | *P*=.61 | 0.02 (−0.44 to 0.47) | *P*=.92 | 0.55 (0.23–0.76) | *P*=.002 |  |
|  |  |  | ND | 0.47 (0.20–0.68) | *P*=.001 | 0.47 (0.27–0.62) | *P*<.001 | 0.21 (−0.28 to 0.62) | *P*=.40 | −0.17 (−0.58 to 0.31) | *P*=.50 | 0.57 (0.29–0.77) | *P*<.001 |  |
| Chorea | UHDRS Maximal Chorea upper limb | Sway path | D | 0.50 (0.23–0.70) | *P*<.001 | 0.46 (0.27–0.62) | *P*<.001 | −0.06 (−0.50 to 0.41) | *P*=.81 | 0.58 (0.17–0.82) | *P*=.010 | 0.47 (0.15–0.70) | *P*=.006 |  |
|  |  |  | ND | 0.58 (0.34–0.75) | *P*<.001 | 0.45 (0.25–0.61) | *P*<.001 | −0.26 (−0.64 to 0.22) | *P*=.29 | 0.27 (−0.22 to 0.66) | *P*=.27 | 0.65 (0.40–0.81) | *P*<.001 |  |
| Balance | Balance score | Sway path | | 0.51 (0.05–0.79) | *P*=.03 | 0.28 (0.05–0.48) | *P*=.02 | −0.20 (−0.62 to 0.30) | *P*=.43 | – | – | 0.24 (−0.16 to 0.56) | *P*=.23 |  |
| U-Turn | TMS | Median turn speed (rad/sec) | | −0.51 (−0.77 to −0.09) | *P*=.01 | −0.16 (−0.38 to −0.07) | *P*=.18 | −0.19 (−0.61 to 0.32) | *P*=.48 | −0.22 (−0.64 to 0.32) | *P*=.45 | −0.20 (−0.55 to 0.20) | *P*=.32 |  |
| Walking | TMS | Step frequency variance (Hz^2^) | | 0.71 (0.42–0.87) | *P*<.001 | 0.26 (0.02–0.47) | *P*=.03 | 0.32 (−0.21 to 0.70) | *P*=.22 | 0.05 (−0.44 to 0.52) | *P*=.85 | 0.47 (0.06–0.72) | *P*=0.02 |  |

^a^Indicates Pearson’s correlation coefficient; Spearman’s correlation coefficients are used otherwise.

^b^The in-clinic Speeded Tapping test was not conducted in the HD NHS and Digital-HD study.
