# Supplementary material for: A Remote Digital Monitoring Platform to Assess Cognitive and Motor Symptoms in Huntington Disease: Cross-sectional Validation Study
Source: J Med Internet Res. 2022 Jun 28;24(6):e32997. doi: 10.2196/32997 (PMC9277525; doi:10.2196/32997)
Supplement: Multimedia Appendix 7 [file jmir_v24i6e32997_app7.docx]

This is a Multimedia Appendix to a full manuscript published in the J Med Internet Res. For full copyright and citation information see <http://dx.doi.org/10.2196/jmir.32997>

Multimedia Appendix 7. *P* values for known-groups validity of digital active tests.

| **Test** | **Digital test feature** | | **Group 1** | **Group 2** | ***P* value** |
| --- | --- | --- | --- | --- | --- |
| SDMT | Number of correct answers | | Digital-HD: Healthy controls | Digital-HD: Premanifest HD | *P*=.09 |
|  |  |  | Digital-HD: Healthy controls | Digital-HD: Manifest HD | *P*<.001 |
|  |  |  | Digital-HD: Healthy controls | HD NHS | *P*<.001 |
|  |  |  | Digital-HD: Healthy controls | OLE study | *P*<.001 |
|  |  |  | Digital-HD: Premanifest HD | Digital-HD: Manifest HD | *P*<.001 |
|  |  |  | Digital-HD: Premanifest HD | HD NHS | *P*<.001 |
|  |  |  | Digital-HD: Premanifest HD | OLE study | *P*=.002 |
|  |  |  | Digital-HD: Manifest HD | HD NHS | *P*<.001 |
|  |  |  | Digital-HD: Manifest HD | OLE study | *P*<.001 |
|  |  |  | HD NHS | OLE study | *P*=.95 |
| SWR | Number of correctly read words | | Digital-HD: Healthy controls | Digital-HD: Premanifest HD | *P*=.48 |
|  |  |  | Digital-HD: Healthy controls | Digital-HD: Manifest HD | *P*<.001 |
|  |  |  | Digital-HD: Healthy controls | HD NHS | *P*<.001 |
|  |  |  | Digital-HD: Healthy controls | OLE study | *P*<.001 |
|  |  |  | Digital-HD: Premanifest HD | Digital-HD: Manifest HD | *P*<.001 |
|  |  |  | Digital-HD: Premanifest HD | HD NHS | *P*=.001 |
|  |  |  | Digital-HD: Premanifest HD | OLE study | *P*=.006 |
|  |  |  | Digital-HD: Manifest HD | HD NHS | *P*<.001 |
|  |  |  | Digital-HD: Manifest HD | OLE study | *P*<.001 |
|  |  |  | HD NHS | OLE study | *P*=.92 |
| Speeded Tapping | Mean inter-tap interval (ms) | D | Digital-HD: Healthy controls | Digital-HD: Premanifest HD | *P*=.23 |
|  |  |  | Digital-HD: Healthy controls | Digital-HD: Manifest HD | *P*<.001 |
|  |  |  | Digital-HD: Healthy controls | HD NHS | *P*=.15 |
|  |  |  | Digital-HD: Healthy controls | OLE study | *P*=.12 |
|  |  |  | Digital-HD: Premanifest HD | Digital-HD: Manifest HD | *P*=.002 |
|  |  |  | Digital-HD: Premanifest HD | HD NHS | *P*=.48 |
|  |  |  | Digital-HD: Premanifest HD | OLE study | *P*=.43 |
|  |  |  | Digital-HD: Manifest HD | HD NHS | *P*=.03 |
|  |  |  | Digital-HD: Manifest HD | OLE study | *P*=.03 |
|  |  |  | HD NHS | OLE study | *P*=.88 |
|  |  | ND | Digital-HD: Healthy controls | Digital-HD: Premanifest HD | *P*=.09 |
|  |  |  | Digital-HD: Healthy controls | Digital-HD: Manifest HD | *P*<.001 |
|  |  |  | Digital-HD: Healthy controls | HD NHS | *P*=.18 |
|  |  |  | Digital-HD: Healthy controls | OLE study | *P*=.14 |
|  |  |  | Digital-HD: Premanifest HD | Digital-HD: Manifest HD | *P*=.003 |
|  |  |  | Digital-HD: Premanifest HD | HD NHS | *P*=.51 |
|  |  |  | Digital-HD: Premanifest HD | OLE study | *P*=.60 |
|  |  |  | Digital-HD: Manifest HD | HD NHS | *P*=.02 |
|  |  |  | Digital-HD: Manifest HD | OLE study | *P*=.02 |
|  |  |  | HD NHS | OLE study | *P*=.82 |
| Draw-A-Shape | Spiral drawing speed variability (mm/s) | D | Digital-HD: Healthy controls | Digital-HD: Premanifest HD | *P*=.08 |
|  |  |  | Digital-HD: Healthy controls | Digital-HD: Manifest HD | *P*<.001 |
|  |  |  | Digital-HD: Healthy controls | HD NHS | *P*<.001 |
|  |  |  | Digital-HD: Healthy controls | OLE study | *P*=.003 |
|  |  |  | Digital-HD: Premanifest HD | Digital-HD: Manifest HD | *P*<.001 |
|  |  |  | Digital-HD: Premanifest HD | HD NHS | *P*=.007 |
|  |  |  | Digital-HD: Premanifest HD | OLE study | *P*=.05 |
|  |  |  | Digital-HD: Manifest HD | HD NHS | *P*=.006 |
|  |  |  | Digital-HD: Manifest HD | OLE study | *P*=.02 |
|  |  |  | HD NHS | OLE study | *P*=.90 |
|  |  | ND | Digital-HD: Healthy controls | Digital-HD: Premanifest HD | *P*=.02 |
|  |  |  | Digital-HD: Healthy controls | Digital-HD: Manifest HD | *P*<.001 |
|  |  |  | Digital-HD: Healthy controls | HD NHS | *P*<.001 |
|  |  |  | Digital-HD: Healthy controls | OLE study | *P*<.001 |
|  |  |  | Digital-HD: Premanifest HD | Digital-HD: Manifest HD | *P*<.001 |
|  |  |  | Digital-HD: Premanifest HD | HD NHS | *P*=.02 |
|  |  |  | Digital-HD: Premanifest HD | OLE study | *P*=.02 |
|  |  |  | Digital-HD: Manifest HD | HD NHS | *P*=.01 |
|  |  |  | Digital-HD: Manifest HD | OLE study | *P*=.02 |
|  |  |  | HD NHS | OLE study | *P*=.89 |
| Chorea | Sway path | D | Digital-HD: Healthy controls | Digital-HD: Premanifest HD | *P*=.24 |
|  |  |  | Digital-HD: Healthy controls | Digital-HD: Manifest HD | *P*<.001 |
|  |  |  | Digital-HD: Healthy controls | HD NHS | *P*<.001 |
|  |  |  | Digital-HD: Healthy controls | OLE study | *P*<.001 |
|  |  |  | Digital-HD: Premanifest HD | Digital-HD: Manifest HD | *P*<.001 |
|  |  |  | Digital-HD: Premanifest HD | HD NHS | *P*=.009 |
|  |  |  | Digital-HD: Premanifest HD | OLE study | *P*=.002 |
|  |  |  | Digital-HD: Manifest HD | HD NHS | *P*=.001 |
|  |  |  | Digital-HD: Manifest HD | OLE study | *P*=.006 |
|  |  |  | HD NHS | OLE study | *P*=.40 |
|  |  | ND | Digital-HD: Healthy controls | Digital-HD: Premanifest HD | *P*=.08 |
|  |  |  | Digital-HD: Healthy controls | Digital-HD: Manifest HD | *P*<.001 |
|  |  |  | Digital-HD: Healthy controls | HD NHS | *P*<.001 |
|  |  |  | Digital-HD: Healthy controls | OLE study | *P*<.001 |
|  |  |  | Digital-HD: Premanifest HD | Digital-HD: Manifest HD | *P*<.001 |
|  |  |  | Digital-HD: Premanifest HD | HD NHS | *P*=.01 |
|  |  |  | Digital-HD: Premanifest HD | OLE study | *P*=.004 |
|  |  |  | Digital-HD: Manifest HD | HD NHS | *P*=.002 |
|  |  |  | Digital-HD: Manifest HD | OLE study | *P*=.02 |
|  |  |  | HD NHS | OLE study | *P*=.56 |
| Balance | Sway path | | Digital-HD: Healthy controls | Digital-HD: Premanifest HD | *P*=.79 |
|  |  |  | Digital-HD: Healthy controls | Digital-HD: Manifest HD | *P*<.001 |
|  |  |  | Digital-HD: Healthy controls | HD NHS | *P*<.001 |
|  |  |  | Digital-HD: Healthy controls | OLE study | *P*<.001 |
|  |  |  | Digital-HD: Premanifest HD | Digital-HD: Manifest HD | *P*<.001 |
|  |  |  | Digital-HD: Premanifest HD | HD NHS | *P*<.001 |
|  |  |  | Digital-HD: Premanifest HD | OLE study | *P*<.001 |
|  |  |  | Digital-HD: Manifest HD | HD NHS | *P*=.08 |
|  |  |  | Digital-HD: Manifest HD | OLE study | *P*=.98 |
|  |  |  | HD NHS | OLE study | *P*=.14 |
| U-Turn | Median turn speed (rad/sec) | | Digital-HD: Healthy controls | Digital-HD: Premanifest HD | *P*=.27 |
|  |  |  | Digital-HD: Healthy controls | Digital-HD: Manifest HD | *P*=.09 |
|  |  |  | Digital-HD: Healthy controls | HD NHS | *P*=.65 |
|  |  |  | Digital-HD: Healthy controls | OLE study | *P*=.81 |
|  |  |  | Digital-HD: Premanifest HD | Digital-HD: Manifest HD | *P*=.51 |
|  |  |  | Digital-HD: Premanifest HD | HD NHS | *P*=.18 |
|  |  |  | Digital-HD: Premanifest HD | OLE study | *P*=.34 |
|  |  |  | Digital-HD: Manifest HD | HD NHS | *P*=.06 |
|  |  |  | Digital-HD: Manifest HD | OLE study | *P*=.16 |
|  |  |  | HD NHS | OLE study | *P*=.99 |
| Walking | Step frequency variance (Hz^2^) | | Digital-HD: Healthy controls | Digital-HD: Premanifest HD | *P*=.17 |
|  |  |  | Digital-HD: Healthy controls | Digital-HD: Manifest HD | *P*<.001 |
|  |  |  | Digital-HD: Healthy controls | HD NHS | *P*=.003 |
|  |  |  | Digital-HD: Healthy controls | OLE study | *P*=.06 |
|  |  |  | Digital-HD: Premanifest HD | Digital-HD: Manifest HD | *P*=.014 |
|  |  |  | Digital-HD: Premanifest HD | HD NHS | *P*=.06 |
|  |  |  | Digital-HD: Premanifest HD | OLE study | *P*=.37 |
|  |  |  | Digital-HD: Manifest HD | HD NHS | *P*=.51 |
|  |  |  | Digital-HD: Manifest HD | OLE study | *P*=.52 |
|  |  |  | HD NHS | OLE study | *P*=.67 |
